# Prevalence of Atherosclerosis-Related Risk Factors and Diseases in the Philippines

**DOI:** 10.2188/jea.JE20110095

**Published:** 2012-09-05

**Authors:** Rody G. Sy, Dante D. Morales, Antonio L. Dans, Elizabeth Paz-Pacheco, Felix Eduardo R. Punzalan, Nelson S. Abelardo, Charmaine A. Duante

**Affiliations:** 1 Section of Cardiology, Department of Medicine, College of Medicine, University of the Philippines (UP) System, Manila, Philippines; 2 Section of Adult Medicine, Department of Medicine, College of Medicine, UP System, Manila, Philippines; 3 Section of Endocrinology, Diabetes and Metabolism, Department of Medicine, College of Medicine, UP System, Manila, Philippines; 4 Food and Nutrition Research Institute, Department of Science and Technology, Taguig, Philippines

**Keywords:** epidemiology, atherosclerosis, risk factors, Philippine data, cardiovascular disease

## Abstract

**Background:**

We conducted a survey in 2008 to measure the prevalence of lifestyle-related diseases and risk factors in Philippine adults.

**Methods:**

Stratified multistage sampling was used to cover the entire Philippine population of adults aged 20 years or older. Using health questionnaires, anthropometric measurements, and blood examinations, the prevalences of atherosclerosis-related risk factors and diseases were determined. Survey results were compared with those obtained in 2003.

**Results:**

Out of 7700 eligible subjects, 64% to 93.7% responded to different survey items. Age-adjusted hypertension prevalence was 24.6% at a single visit and 20.6% when corrected for true prevalence. The prevalence of diabetes was 3.9% on the basis of fasting blood glucose (FBG), 5.2% by FBG and history, and 6.0% when 2-hour post-load plasma glucose level was determined. The prevalence of dyslipidemia was 72.0% and the prevalence of smoking was 31%. The prevalence of obesity was 4.9% by body mass index (BMI), and 10.2% and 65.6% by waist-hip ratio (WHR) in men and women, respectively. The prevalences of coronary, cerebrovascular, and peripheral arterial diseases were 1.1%, 0.9%, and 1.0%, respectively.

**Conclusions:**

The prevalences of risk factors for atherosclerosis were higher in 2008 than in 2003, although the increase in diabetes was not significant and smoking decreased. These findings indicate a need for active collaborative intervention by all government agencies and medical societies in the Philippines.

## INTRODUCTION

Cardiovascular disease has been the number one cause of mortality in the Philippines for many years.^1^ The first national survey of the prevalence of atherosclerosis-related risk factors and diseases (hypertension, diabetes, dyslipidemia, obesity, smoking, angina, and stroke) among adults was done in 1998.^2^ In 2003, another national survey was conducted and expanded the scope of the research to other noncommunicable or lifestyle-related condition; a total of 32 conditions were reported in what was now referred to as the National Nutrition and Health Survey I (NNHeS I).^3^ The methods of the 2 surveys differed, specifically with regard to blood collection and analysis, but the prevalence rates were similar.

In 2008, the Department of Science and Technology—Food and Nutrition Research Institute (DOST-FNRI) and the Department of Health (DOH) conducted the latest national nutrition survey and collaborated with 12 medical societies for the clinical and health component. The survey examined the prevalences of the same risk factors and diseases and utilized the same blood specimen and laboratory analysis techniques used in 2003. This new study is referred to as the National Nutrition and Health Survey II (NNHeS II): 2008. This article will report the prevalences of 5 atherosclerosis risk factors (hypertension, diabetes, dyslipidemia, obesity, and smoking) and 3 atherosclerosis-related diseases—coronary artery disease (CAD), cerebrovascular disease (CVD), and peripheral arterial disease (PAD). Because NNHeS II is very similar to NNHeS I, this report will also compare the prevalence data between the 2 surveys.

The report aims to present an accurate picture of the current cardiovascular health of the Philippine population and guide the future actions of government health agencies and professional health organizations.

## METHODS

The method of this survey was similar to that used in NNHeS I: 2003, which has been described previously.^4^ The study protocol was approved by the Technical Committee and Ethics Review Board of the Department of Science and Technology. Briefly, this survey used the National Statistics Office (NSO) 2008 Labor Force Survey (LFS) master sample and employed a stratified multi-stage sampling design to represent each of the 17 regions of the country. The first stage was the selection of primary sampling units (PSUs) in a *barangay* (household unit) or contiguous *barangays* with at least 500 households, with a probability proportional to the estimated number of households. The second stage was the selection of enumeration areas (EAs) within the sampled PSUs, with 150 to 200 households serving as the secondary sampling units (SSUs). The last stage was the selection of housing units within the sampled EAs, which served as the ultimate sampling unit (USU). As such, the household was considered a cluster in which all the units became part of the survey. The clinical component included adults aged 20 years or older. All participants gave written informed consent for the survey.

The study used standardized methods of anthropometric, clinical, and health assessment for data collection. Face-to-face interviews were conducted to obtain pertinent medical information and answers to questionnaires, which included a prior diagnosis by medical doctor or nurse of CAD (previous heart attack or documented ischemia or coronary occlusive disease; not angina symptoms), cerebrovascular accident (previous stroke or transient ischemic attack), or PAD (previously diagnosed occlusive disease). Field workers underwent comprehensive training before they were deployed. Anthropometric data collected included height, weight, waist circumference (WC), and waist-hip ratio (WHR); body mass index (BMI) was calculated using the standard formula. Variability in measurements, specifically those for the waist and hips, was assessed by reliability testing among the data collectors during training before actual field collection of data. Blood pressure (BP) was measured using a mercury sphygmomanometer (Baumanometer), and the mean of 3 measurements taken during a single visit was used in the analysis. The respondents were requested to rest for at least 5 minutes before BP measurement. All field researchers were instructed to take BP readings before blood extraction to ensure that respondents had not consumed any beverages, as they were required to fast. Participants were also instructed not to smoke before BP measurement. After a 10- to 12-hour fast, blood samples were drawn by trained, registered medical technologists. Analysis of fasting blood glucose (FBG), total cholesterol (TC), triglycerides (TG), low-density lipoprotein cholesterol (LDL-C; obtained by computation), and high-density lipoprotein cholesterol (HDL-C) was done in a central laboratory using a Roche Cobas Mira blood analyzer with recommended reagents and with results validated by the Clinical Chemistry Division, Department of Pathology, Ramathibodi Hospital, Mahidol University in Bangkok, Thailand. This laboratory is accredited by the US Centers for Disease Control (CDC) in Atlanta, USA. Glucose levels were also measured 2 hours after participants consumed a standard 75-g glucose load.

NNHeS I in 2003 also used a stratified multistage sampling design that utilized the master sample of the Family Income and Expenditures Survey (FIES) of the NSO. Sampling was done at the *barangay*/community, EA, and household levels, and all members in the sampled households were included in the survey. The parameters measured and questionnaires administered were identical to those in the NNHes II Survey, with the exception of 2-hour post-load plasma glucose level and other additional laboratory and clinical tests, which were included in the 2008 survey.^3^ Glucose and lipid measurements were done in the same central laboratory in Manila, and the lipid values were validated by the US Veterans Administration Hospital, which is accredited by the American Association of Clinical Pathologists.

### Operational definitions

High blood pressure (hypertension) was defined, according to JNC-7 recommendations, as systolic BP (SBP) of 140 mm Hg or higher, diastolic BP (DBP) of 90 mm Hg or higher, a previous diagnosis of hypertension, or use of antihypertensive medication.^5^ The first Korotkoff sound was used to record SBP, and the fifth Korotkoff sound was used to record DBP. True prevalence of hypertension was determined by using the Marchevsky equation, as recommended for calculating real prevalence in population studies. The equation used is P = A − (100% − Sp) / Sn − (100% − Sp), where P is the estimated true prevalence in percent, A is the crude prevalence in percent as determined by the survey instrument, and Sn and Sp are its sensitivity and specificity.^6^^,^^7^

Diabetes was defined as a fasting plasma glucose level of 7.0 mmol/L (126 mg/dL) or higher, a 2-hour post-load plasma glucose level of 11.1 mmol/L (200 mg/dL) or higher,^8^ or a history of diabetes, with or without use of oral antihyperglycemic medication or insulin. We did not differentiate between type 1 and type 2 diabetes.

Body mass index (BMI) was calculated as the weight in kilograms divided by the square of the height in meters. BMI was classified according to the following scale: less than 18.5 kg/m^2^ was defined as chronic energy deficiency (CED), 18.5 to 24.9 kg/m^2^ was defined as normal, 25.0 to 29.9 kg/m^2^ was defined as overweight, and 30 kg/m^2^ or higher was defined as obese.^9^

WHR was measured by dividing waist circumference (measured at the midpoint between the lowest rib and the iliac crest) by the hip circumference (measured at the greater trochanter or widest diameter of the hips). Obesity was defined as a WHR of 1.0 or higher for men and 0.85 or higher for women.^10^

The cutoffs used for blood lipids were consistent with those used in the previous 2003 NNHeS study and in the Third Report of the National Cholesterol Education Program-Adult Treatment Panel III (NCEP-ATP III).^11^ The definitions of high TC, high LDL-C, and high TG were 6.20 mmol/L (240 mg/dL) or higher, 4.14 mmol/L (160 mg/dL) or higher, and 2.26 mmol/L (200 mg/dL) or higher, respectively. Low HDL-C was defined as less than 1.03 mmol/L (40 mg/dL). An abnormal value for any of these parameters was defined as dyslipidemia.

Smoking status was classified using the 3 World Health Organization (WHO) categories: never-smoker for those who have never tried smoking tobacco or cigarettes, former smoker for those who tried but did not continue using tobacco or cigarettes, or current smoker for those who were still smoking tobacco or cigarettes on the survey day.

### Data organization, editing, processing, and analysis

Data collected in the field were checked for completeness. Data were entered by hired data encoders using a MySQL database. Two rounds of proofreading were done to ensure the correctness and validity of encoded data. After manual and computerized data validation, several data files were merged to create a master dataset, which was followed by another round of checking and validation of the dataset to eliminate errors and inconsistencies. Sampling weights were computed and attached to the master dataset in preparation for data analysis. Stata software Release 11 (Serial No. 196048004) was used to process and analyze the clinical and health parameters to identify the distributions of disease and risk factors according to age, sex, and urban classification.

Sampling designs other than simple random sampling can result in bias and greater variance of the estimator. Bias due to the multistage sampling design used in this study was adjusted for by including sample weighting factors in the statistical analyses to compensate for unequal probabilities of selection, noncoverage of the population, and nonresponse. Construction of the overall weighting factor included 3 preliminary weights: sampling weight, base weight (weighting for unequal selection probability), and weight for nonresponse adjustment. Before this activity, eligible and ineligible respondents were identified. Eligible respondents were those who were available at the time of the survey, even if they refused to participate or did not provide the requested information. These individuals were categorized as nonresponders. Those who were not present during data collection (eg, vacant or demolished housing units, transfers) were considered ineligible respondents. Due to the high cost of laboratory analyses, the study included only 25% of the total sample households in an enumeration area (EA); thus, the sampling weight was equal to 8. Base weight was computed by multiplying the original base weight provided by National Statistics Office (NSO) with the sampling weight. Weighted eligibility and weighted response were computed by multiplying the base weight by the number of eligible respondents and number of responses, respectively. Next, strata were formed based on the similarity of characteristics with varied size depending on the number of EAs with similar characteristics within a province. Strata were also referred to as weighting cells or weighting classes. Weighted response rate in a particular stratum/weighting cell/class was computed. The reciprocal of the weighted response was the nonresponse weight. The overall weight of each EA was the product of the base weight and the nonresponse weight. All estimates presented in this study are weighted estimates of adequate precision for the national level.^12^

Population means and rates of some risk factors for atherosclerotic diseases were adjusted to account for the effect of age and to allow comparison of 2 populations at 2 different survey periods. Age standardization was done by creating age groups (in decades) with the corresponding age-adjustment weights using the 2000 Philippine Census of Population. Age-adjustment weights for each of age distribution were obtained by dividing the age-specific counts by the total population over all ages in the age distribution. Age-adjustment factors were incorporated in the analysis using Stata v. 11. Crude and age-adjusted population means and rates for 2003 and 2008 were compared and corresponding *P* values for the differences are presented.

## RESULTS

### Response rate and characteristics of respondents

The clinical component recruited a total of 3744 households from 79 provinces with 3377 EAs and 7700 eligible subjects. The response rates for blood pressure measurement, blood collection for determination of fasting glucose, 2-hour post-load plasma glucose, and fasting lipid parameters, and questionnaire participation were 92.8%, 80.2%, 64.0%, 81.5%, and 93.7% respectively (eTable 1). The highest response rate was for the questionnaire and the lowest response was for 2-hour post-load plasma glucose level (36% refused the test or failed to return for the second blood sample).

A total of 7215 eligible adults aged 20 years or older responded to the questionnaires; 53.9% were women and 46.1% were men (Table 1A); and 52.6% lived in urban areas, while 47.4% lived in rural areas. Post-stratification age adjustment was done to account for the effects of age on all age-dependent variables. Nonresponders were 2.3 years younger than responders, while the proportion of male nonresponders was higher than that of female nonresponders for the BP and questionnaire sections of the survey (Table 1B). Table 1A shows the characteristics of the respondents in the 2 surveys; 58.5% of respondents were 50 years or older in 2003, while only 31.7% of respondents were in this age category in 2008. Participants were not classified as urban or rural dwellers in 2003.

**Table 1A.** Profiles of respondents in 2003 and 2008

Age group, y	2003			2008		
	
	Men	Women	All	Men	Women	All
20–29	389 (17.2)	310 (12.4)	699 (14.7)	812 (24.4)	922 (23.7)	1734 (24.0)
30–39	389 (17.2)	316 (12.6)	705 (14.8)	760 (22.9)	891 (22.9)	1651 (22.9)
40–49	287 (12.7)	281 (11.2)	568 (12.0)	748 (22.5)	801 (20.6)	1549 (21.5)
50–59	204 (9.0)	249 (10.0)	453 (9.5)	494 (14.9)	608 (15.6)	1102 (15.3)
60–69	613 (27.2)	780 (31.2)	1393 (29.3)	314 (9.4)	398 (10.2)	712 (9.9)
≥70	373 (16.5)	562 (22.5)	935 (19.7)	195 (5.9)	272 (7.0)	467 (6.5)
Total	2255 (47.4)	2498 (52.6)	4753	3323 (46.1)	3892 (53.9)	7215

Values are numbers (%).

**Table 1B.** Profile of responders and nonresponders in 2008 survey

Parameter	Responders	Nonresponders	*P*-value
Blood Pressure			
Mean age, y	42.6	40.3	0.0007
Sex			
Male, %	45.5	54.2	0.0035
Female, %	54.5	45.8	0.0077
Blood lipids			
Mean age, y	43.4	38.4	0.0000
Sex			
Male, %	46.9	43.8	0.1725
Female, %	53.1	56.2	0.1211
FBG			
Mean age, y	43.5	38.3	0.0000
Sex			
Male, %	46.8	44.3	0.2430
Female, %	53.2	55.7	0.1882
Questionnaires			
Mean age, y	42.6	40.3	0.0003
Sex			
Male, %	45.4	58.5	0.0000
Female, %	54.6	41.5	0.0003

Abbreviation: FBG, fasting blood glucose.

### Prevalence of atherosclerosis risk factors

#### Hypertension

The overall age-adjusted prevalence of hypertension based on measurements at a single visit was 24.6% (28.4% for men and 21.4% for women), while the true prevalence of hypertension was 20.6% (24.1% for men and 17.4% for women) (Tables 2A and 2B), which was an increase from the prevalence of 16.4% found in the 2003 survey. Among participants identified as hypertensive during the interview, 40.9% were on antihypertensive medication in 2008, as compared with 35.4% in 2003. Based on the JNC VII BP classification, there were more participants in the prehypertensive category in 2003 than in 2008; the reverse was true for the hypertensive category (eTables 2A and 2B).

**Table 2A.** Unadjusted prevalence (%) of atherosclerosis-related risk factors and diseases by sex (Philippines, 2003 and 2008)

	2003	2008	Crude *P*-value
	
	% (95% CI)	% (95% CI)	
**Men**			
Hypertension, single visit	24.2 (23.2, 25.2)	29.1 (26.7, 31.6)	0.0217
Hypertension, true prevalence	18.4 (16.9, 19.9)	28.3 (26.8, 29.8)	0.0000
Diabetes by FBG	3.2 (2.5, 3.8)	4.0 (3.2, 4.8)	0.7737
Diabetes by FBG or history	4.1 (3.3, 4.9)	5.9 (5.0, 6.8)	0.4919
Diabetes by FBG, history or 2-hr PG load	N.A.	7.0 (6.1, 8.0)	N.A.
Dyslipidemia	67.0 (65.2, 68.8)	78.8 (77.2, 80.4)	0.0000
Smoking	57.4 (55.1, 59.7)	53.2 (51.1, 54.8)	0.1206
Obesity by BMI (≥30)	3.2 (2.5, 3.8)	3.7 (3.5, 3.9)	0.8639
Obesity by WHR (≥1.0)	12.1 (10.4, 13.7)	11.1 (9.7, 12.5)	0.6572
CAD	1.2 (1.2, 1.2)	1.3 (0.7, 1.7)	0.9669
Cerebrovascular disease (stroke)	1.3 (1.0, 1.6)	1.6 (1.1, 2.0)	0.3622
PAD	0.2 (0.03, 0.4)	0.9 (0.6, 1.2)	0.8839
**Women**			
Hypertension, single visit	20.8 (19.8, 21.8)	22.2 (20.1, 24.3)	0.4604
Hypertension, true prevalence	16.5 (15.0, 18.0)	23.5 (22.0, 24.8)	0.0000
Diabetes by FBG	3.5 (2.8, 4.2)	5.5 (4.6, 6.3)	0.4444
Diabetes by FBG or history	5.0 (4.2, 5.8)	6.4 (5.6, 7.3)	0.5452
Diabetes by FBG, history, or 2-hr PG load	N.A.	7.4 (6.4, 8.3)	N.A.
Dyslipidemia	57.8 (56.0, 59.6)	68.1 (66.2, 70.0)	0.0000
Smoking	10.9 (9.4, 12.4)	12.5 (11.2, 13.6)	0.4684
Obesity by BMI (≥30)	6.6 (5.9, 7.2)	6.6 (6.4, 6.8)	1.0000
Obesity by WHR (≥0.85)	54.8 (51.5, 58.0)	65.5 (65.1, 65.9)	0.0000
CAD	2.4 (1.6, 3.2)	1.4 (1.0, 1.8)	0.6951
Cerebrovascular disease (stroke)	1.5 (1.2, 1.8)	1.0 (0.6, 1.3)	0.8283
PAD	0.6 (0.3, 0.9)	1.5 (1.1, 1.9)	0.7988
**All**			
Hypertension, single visit	22.5 (21.5, 24.7)	25.3 (23.6, 27.1)	0.0487
Hypertension, true prevalence	17.4 (15.9, 18.9)	25.7 (24.5, 26.8)	0.0000
Diabetes by FBG	3.4 (2.7, 4.0)	4.8 (4.2, 5.4)	0.4494
Diabetes by FBG or history	4.6 (3.9, 5.3)	6.2 (5.6, 6.9)	0.3561
Diabetes by FBG, history, 2-hr PG load	N.A.	7.2 (6.5, 7.9)	N.A.
Dyslipidemia	62.5 (60.7, 64.3)	73.1 (71.8, 74.5)	0.0000
Smoking	35.2 (33.7, 36.7)	31.0 (29.7, 32.0)	0.0061
Obesity by BMI (≥30)	4.8 (4.1, 6.8)	5.2 (5.0, 5.4)	0.8445
CAD	1.8 (1.3, 2.3)	1.3 (1.0, 1.6)	0.7720
Cerebrovascular (stroke)	1.4 (1.1, 1.7)	1.2 (1.0, 1.5)	0.3408
PAD	0.4 (0.2, 0.6)	1.2 (0.9, 1.5)	0.7690

Abbreviations: FBG, fasting blood glucose; 2-hr PG load, 2-hour post-load plasma glucose level; BMI, body mass index; WHR, waist-hip ratio; CAD, coronary artery disease; PAD, peripheral artery disease.

**Table 2B.** Age-adjusted prevalence (%) of atherosclerosis-related risk factors and diseases by sex (Philippines 2003 and 2008)

	2003	2008	Age adj *P*-value
	
	% (95% CI)	% (95% CI)	
**Men**			
Hypertension, single visit	24.5 (21.4, 27.6)	28.4 (25.7, 31.1)	0.0670
Hypertension, true prevalence	19.0 (16.6, 21.4)	24.1 (22.9, 25.3)	0.0000
Diabetes by FBG	3.4 (0.6, 7.4)	3.3 (0.6, 6.7)	0.9701
Diabetes by FBG or history	4.5 (3.4, 5.5)	4.9 (1.8, 8.0)	0.7775
Diabetes by FBG, history, or 2-hr PG load	N.A.	5.9 (5.0, 6.7)	N.A.
Dyslipidemia	67.0 (64.6, 69.4)	79.0 (77.4, 80.6)	0.0000
Smoking	56.5 (53.6, 59.3)	53.9 (51.6, 56.2)	0.1681
Obesity by BMI (≥30)	3.1 (1.2, 7.4)	3.6 (0.2, 7.0)	0.8621
Obesity by WHR (≥1.0)	12.0 (10.4, 13.7)	10.2 (9.3, 11.2)	0.4411
CAD	1.3 (−2.0, 4.6)	1.0 (−2.0, 4.1)	0.8974
Cerebrovascular disease (stroke)	1.4 (−1.2, 4.0)	1.2 (−1.8, 4.2)	0.9228
PAD	0.2 (−4.2, 4.6)	0.8 (−2.4, 4.0)	0.8946
**Women**			
Hypertension, single visit	18.4 (19.8, 21.8)	21.4 (18.9, 23.9)	0.1040
Hypertension, true prevalence	12.9 (10.9, 14.9)	17.4 (16.0, 18.8)	0.0000
Diabetes by FBG	3.3 (−0.05, 6.6)	4.3 (1.2, 7.3)	0.6741
Diabetes by FBG or history	4.3 (3.3, 5.3)	5.4 (2.6, 8.2)	0.6077
Diabetes by FBG, history, or 2-hr PG load	N.A.	6.0 (5.3, 6.8)	N.A.
Dyslipidemia	57.1 (54.6, 59.6)	66.0 (64.0, 68.0)	0.0000
Smoking	11.4 (8.4, 14.4)	11.7 (8.8, 14.6)	0.8874
Obesity by BMI (≥30)	6.3 (2.0, 10.6)	6.1 (2.7, 9.5)	0.9426
Obesity by WHR (≥1.0)	53.0 (50.0, 56.0)	65.6 (63.9, 67.3)	0.0000
CAD	2.1 (–0.8, 5.0)	1.2 (−1.9, 4.3)	0.7058
Cerebrovascular disease (stroke)	1.3 (−1.2, 3.8)	0.7 (−2.0, 3.4)	0.7752
PAD	1.0 (−4.4, 6.4)	1.3 (−1.6, 4.2)	0.9296
**All**			
Hypertension, single visit	21.6 (19.6, 23.6)	24.6 (22.7, 26.5)	0.0327
Hypertension, true prevalence	16.4 (14.8, 18.0)	20.6(19.4, 21.8)	0.0000
Diabetes by FBG	3.3 (0.7, 5.9)	3.9 (1.6, 6.2)	0.7354
Diabetes by FBG or history	4.4 (3.7, 5.2)	5.2 (3.1, 7.3)	0.7151
Diabetes by FBG, history, or 2-hr PG load	N.A.	6.0 (5.4, 6.6)	N.A.
Dyslipidemia	62.3 (60.5, 64.0)	72.0 (70.7, 73.3)	0.0000
Smoking	35.9 (33.6, 38.2)	31.0 (29.1, 32.9)	0.0013
Obesity by BMI (≥30)	4.7 (1.6, 7.7)	4.9 (2.5, 7.3)	0.9202
CAD	1.7 (−0.4, 3.9)	1.1 (−1.1, 3.3)	0.7161
Cerebrovascular disease (stroke)	1.4 (−0.4, 3.2)	0.9 (−1.1, 2.9)	0.7337
PAD	0.6 (−3.1, 4.3)	1.0 (−1.0, 3.0)	0.8752

Abbreviations: FBG, fasting blood glucose; 2-hr PG load, 2-hour post-load plasma glucose level; BMI, body mass index; WHR, waist-hip ratio; CAD, coronary artery disease; PAD, peripheral artery disease.

Age-adjusted mean SBP and DBP in 2008 were 120.2 and 78.6 mm Hg overall, 122.8 and 80.7 mm Hg for men, and 118.0 and 76.7 mm Hg for women. In 2003, the corresponding overall age-adjusted mean SBP and DBP were 121.0 and 76.6 mm Hg, respectively (Tables 3A and 3B).

**Table 3A.** Unadjusted population means for atherosclerosis-related risk factors and diseases by sex (Philippines 2003 and 2008)

	2003	2008	Unadjusted *P*-value
	
	Mean (95% CI)	Mean (95% CI)	
**Men**			
Systolic blood pressure, mm Hg	123.1 (122.2, 124.1)	123.0 (122.3, 123.7)	0.8619
Diastolic blood pressure, mm Hg	78.4 (77.8, 79.1)	80.8 (80.3, 81.3)	0.0000
Fasting blood glucose, mmol/L	4.45 (4.36, 4.53)	4.85 (4.78, 4.92)	0.0000
Total cholesterol, mmol/L	4.63 (4.58, 4.67)	4.66 (4.62, 4.70)	0.2138
LDL cholesterol, mmol/L	2.91 (2.86, 2.95)	2.84 (2.81, 2.88)	0.0003
HDL cholesterol, mmol/L	1.04 (1.03, 1.05)	1.01 (1.00, 1.02)	0.0972
Triglyceride, mmol/L	1.47 (1.42, 1.52)	1.75 (1.71, 1.79)	0.0000
BMI	22.5 (22.3, 22.7)	22.6 (22.4, 22.7)	0.2948
Waist circumference, cm	79.3 (78.7, 79.9)	79.4 (79.1, 79.8)	0.7282
Waist-hip ratio	0.91 (0.906, 0.914)	0.92 (0.920, 0.925)	0.0000
**Women**			
Systolic blood pressure, mm Hg	119.7 (118.5 120.9)	118.7 (118.0, 119.4)	0.1073
Diastolic blood pressure, mm Hg	75.3 (74.6, 76.0)	77.0 (76.6, 77.5)	0.0000
Fasting blood glucose, mmol/L	4.51 (4.41, 4.61)	4.93 (4.85, 5.01)	0.0000
Total cholesterol, mmol/L	4.92 (4.86, 4.98)	4.98 (4.94, 5.03)	0.0342
LDL cholesterol, mmol/L	3.28 (3.23, 3.33)	3.25 (3.21, 3.29)	0.0470
HDL cholesterol, mmol/L	1.10 (1.10, 1.11)	1.06 (1.06, 1.07)	0.0476
Triglyceride, mmol/L	1.18 (1.15, 1.21)	1.46 (1.43, 1.49)	0.0000
BMI	23.0 (22.7, 23.2)	23.1 (22.8, 23.1)	0.6605
Waist circumference, cm	76.8 (76.2, 77.5)	78.1 (77.8, 78.5)	0.0000
Waist-hip ratio	0.85 (0.850, 0.859)	0.87 (0.870, 0.880)	0.0000
**All**			
Systolic blood pressure, mm Hg	121.5 (120.7, 122.7)	120.7 (120.1, 121.25)	0.0606
Diastolic blood pressure, mm Hg	76.8 (76.3, 77.4)	78.8 (78.4, 79.1)	0.0000
Fasting blood glucose, mmol/L	4.47 (4.41, 4.55)	4.89 (4.83, 4.95)	0.0000
Total cholesterol, mmol/L	4.77 (4.73, 4.81)	4.83 (4.79, 4.87)	0.0024
LDL cholesterol, mmol/L	3.09 (3.05, 3.13)	3.06 (3.03, 3.09)	0.0001
HDL cholesterol, mmol/L	1.07 (1.06, 1.08)	1.04 (1.03, 1.04)	0.0160
Triglyceride, mmol/L	1.33 (1.30, 1.36)	1.60 (1.57, 1.62)	0.0000
BMI	22.7 (22.5, 22.9)	22.9 (22.6, 22.9)	0.3685

Abbreviations: LDL, low-density lipoprotein; HDL, high-density lipoprotein; BMI, body mass index.

**Table 3B.** Age-adjusted population means for atherosclerosis-related risk factors and diseases by sex, (Philippines 2003 and 2008)

	2003	2008	Adjusted *P*-value
	
	Mean (95% CI)	Mean (95% CI)	
**Men**			
Systolic blood pressure, mm Hg	123.5 (122.6, 124.4)	122.8 (122.2, 123.6)	0.2233
Diastolic blood pressure, mm Hg	78.4 (77.7, 79.0)	80.7 (80.2, 81.1)	0.0000
Fasting blood glucose, mmol/L	4.46 (4.37, 4.54)	4.76 (4.71, 4,82)	0.0000
Total cholesterol, mmol/L	4.63 (4.58, 4.68)	4.60 (4.57, 4.65)	0.5661
LDL cholesterol, mmol/L	2.91 (2.86, 2.96)	2.80 (2.77, 2.84)	0.0422
HDL cholesterol, mmol/L	1.04 (1.03, 1.05)	1.01 (1.00, 1.02)	0.0972
Triglyceride, mmol/L	1.47 (1.42, 1.52)	1.74 (1.70, 1.78)	0.0000
BMI	22.4 (22.2, 22.6)	22.4 (22.3, 22.5)	1.0000
Waist circumference, cm	79.3 (78.7, 79.9)	79.0 (78.7, 79.4)	0.9910
Waist-hip ratio	0.91 (0.906, 0.913)	0.92 (0.918, 0.922)	0.0000
**Women**			
Systolic blood pressure, mm Hg	117.9 (117.0, 118.8)	118.0 (117.4, 118.6)	0.8720
Diastolic blood pressure, mm Hg	74.6 (74.0, 75.2)	76.7 (76.3, 77.2)	0.0000
Fasting blood glucose, mmol/L	4.47 (4.37, 4.56)	4.81 (4.74, 4.88)	0.0000
Total cholesterol, mmol/L	4.88 (4.83, 4.94)	4.85 (4.82, 4.89)	0.1580
LDL cholesterol, mmol/L	3.25 (3.20, 3.30)	3.14 (3.11, 3.17)	0.0052
HDL cholesterol, mmol/L	1.10 (1.09, 1.11)	1.06 (1.05, 1.07)	0.0476
Triglyceride, mmol/L	1.16 (1.13, 1.20)	1.41 (1.38, 1.43)	0.0000
BMI	22.9 (22.6, 23.1)	22.8 (22.6, 22.9)	0.9920
Waist circumference, cm	76.3 (75.7, 76.9)	77.2 (76.9, 77.6)	0.0009
Waist-hip ratio	0.85 (0.847, 0.855)	0.87 (0.871, 0.875)	0.0000
**All**			
Systolic blood pressure, mm Hg	121.0 (120.3, 121.7)	120.2 (119.7, 120.7)	0.0606
Diastolic blood pressure, mm Hg	76.6 (76.2, 77.1)	78.6 (78.2, 78.9)	0.0000
Fasting blood glucose, mmol/L	4.47 (4.40, 4.53)	4.79 (4.74, 4.83)	0.0000
Total cholesterol, mmol/L	4.76 (4.72, 4.80)	4.74 (4.71, 4.77)	0.2058
LDL cholesterol, mmol/L	3.08 (3.05, 3.12)	2.98 (2.95, 3.01)	0.0021
HDL cholesterol, mmol/L	1.07 (1.06, 1.08)	1.04 (1.03, 1.04)	0.0160
Triglyceride, mmol/L	1.33 (1.30, 1.36)	1.57 (1.54, 1.60)	0.0000
BMI	22.7 (22.5, 22.8)	22.6 (22.5, 22.7)	0.1563

Abbreviations: LDL, low-density lipoprotein; HDL, high-density lipoprotein; BMI, body mass index.

#### Diabetes

In 2008, age-adjusted prevalence of diabetes based on FBG was 3.9% overall, 3.3% for men, and 4.3% for women. Overall prevalence increased from 3.3% in 2003. In 2003, the true prevalence of diabetes, based on FBG and questionnaire responses, was 4.4% (4.5% for men and 4.3% for women). In 2008, this increased to 5.2% (4.9% for men and 5.4% for women). When 2-hour post-load plasma glucose level was added as a criterion, the true prevalence increased to 6.0% (5.9% for men and 6.0% for women; Tables 2A and 2B). Among participants who reported having diabetes during the interview, 52.9% in 2003 and 51.6% in 2008 were on antidiabetic medication. No distinction was made between type 1 and type 2 diabetes in either survey.

Age-adjusted mean FBG in 2008 was 4.79 mmol/L (4.76 mmol/L for men and 4.81 mmol/L for women). In 2003, the corresponding mean FBG was 4.47 mmol/L (4.46 mmol/L for men and 4.47 mmol/L for women; Tables 3A and 3B).

#### Dyslipidemia

Dyslipidemia was defined as high TC, TG, or LDL-C or low HDL-C. The age-adjusted prevalence of dyslipidemia in 2008 was 72.0% overall, 79.0% for men, and 66.0% for women. The prevalence of dyslipidemia in 2003 was 62.3% (Tables 2A and 2B). In both surveys, the high prevalence of dyslipidemia was mainly caused by the high prevalence of low HDL-C. In 2008, the prevalence of low HDL-C was 64.1%, whereas the prevalences of high triglycerides, high LDL-C, and high total cholesterol were 14.6%, 11.8%, and 10.2%, respectively. Low TC (<4.14 mmol/L) was observed in 28.8% of participants in 2003 and 28.3% of those in 2008 (eTable 3).

In 2008, the age-adjusted mean values for total cholesterol, LDL-C, HDL-C, and triglycerides were 4.74, 2.98, 1.04, and 1.5 mmol/L, respectively. Except for triglyceride level, which is typically higher in men, the lipid parameters were all higher in women. Mean values for total cholesterol, LDL-C, HDL-C, and triglycerides in 2003 were 4.76, 3.08, 1.07, and 1.33 mmol/L, respectively (Tables 3A and 3B).

#### Smoking

The age-adjusted prevalence of smoking in 2008 was 31.0% (53.9% for men and 11.7% for women), as compared with 35.9% in 2003 (56.5% for men and 11.4% for women). Overall prevalence was lower in 2008, reflecting an overall decrease of 13.6% that was mainly due to a 4.6% decline in the proportion of male smokers. However, there was a 2.6% increase in female smokers (Table 2B).

#### Obesity

Using BMI criteria based on WHO guidelines, the age-adjusted prevalence of obesity in 2008 was 4.9% (3.6% for men and 6.1% for women), a minimal increase from the 2003 prevalence of 4.7% (3.1% for men and 6.3% for women). Mean BMI for the population was 22.6 kg/m^2^ in 2008 and 22.7 kg/m^2^ in 2003 (Tables 2A and 2B). Respondents in the CED category were 11.6% in 2008 and 12.4% in 2003. The prevalence of overweight/obesity (BMI ≥25) was 26.6% in 2008 and 24.4% in 2003 (eTables 4A and 4B).

When obesity was defined as a WHR of 1.0 or higher, the age-adjusted prevalence of obesity in men was 10.2% in 2008 and 12.0% in 2003. With the lower cut-off of 0.85 or higher in women, the prevalence was 65.6% in 2008 and 53.0% in 2003 (Table 2B).

In 2008, the age-adjusted mean WHR was 0.92 in men and 0.87 in women as compared with 0.91 in men and 0.85 in women in 2003. Mean WC was 79.0 cm in men and 77.2 cm in women in 2008 as compared with 79.3 cm in men and 76.3 cm in women in 2003 (Tables 3A and 3B).

On the basis of the harmonized guidelines for metabolic syndrome,^13^ the prevalence of metabolic syndrome was 18.6% in 2003 and 27.4% in 2008. In both periods, the highest component was low HDL-C, followed by elevated BP and increased WC. Each of the 5 metabolic risk factors increased in prevalence from 2003 to 2008 (eTable 5). Our findings regarding the metabolic syndrome will be reported in a future article.

### Prevalence of atherosclerosis-related diseases

The prevalence estimates for the 3 atherosclerosis-related diseases (CAD, cerebrovascular disease, and PAD) were based on previous diagnoses of the conditions by medical personnel, as indicated on interview questionnaires. The overall age-adjusted prevalence rates in 2008 were 1.1%, 0.9%, and 1.1% respectively. The corresponding rates in 2003 were 1.1%, 1.4%, and 0.6% respectively (Tables 2A and 2B).

## DISCUSSION

Although mean blood pressure did not vary much over the past 5 years, the true prevalence of hypertension in this survey was 20.6% in the overall population, which is a 4.2% absolute increase as compared with the 16.4% prevalence in 2003.^3^ This suggests that efforts to prevent and control hypertension have been insufficient. The increased true prevalence may also be due to poor BP control, as 40.9% of those with self-reported hypertension were on medication in 2008 as compared with 35.4% in 2003.

The prevalence of diabetes, based on FBG alone, increased during the last 5 years, although the change was not significant. This was corroborated by an increase in the true prevalence of diabetes (based on FBG and questionnaire responses) from 4.4% in 2003 to 5.2% in 2008, which was also nonsignificant. Inclusion of 2-hour post-load plasma glucose level in the criteria for diabetes increased true prevalence to 6.0%. Among those with self-reported diabetes, 52.9% in 2003 and 51.6% in 2008 were on antidiabetic medication. This increasing trend in the Philippines is consistent with the rising prevalence of diabetes in Southeast Asia, as well as in Western countries.^14^ It is important to note that we made no distinction between type 1 and type 2 diabetes.

The prevalence of dyslipidemia was 72.0% in 2008 and 62.3% in 2003. This 15.6% relative increase was significant. These high prevalences are principally due to the high prevalence of low HDL-C in both surveys. Triglycerides were higher in men than in women, while the other 3 lipid parameters were higher in women. The US NHANES data showed a dyslipidemia prevalence of 48%, but the cutoff for low HDL-C was different for men and women (<1.04 and <1.3 mmol/L, respectively); thus, the reported figure would likely be lower if the same, lower HDL-C cut-off was used for both sexes.^15^

There was a net decrease of 13.6% in the prevalence of smoking. The decrease was entirely due to a reduction in the percentage of male smokers, as there was an increase of female smokers. The overall smoking rate in the country remains high as compared to the reduced rates in some neighboring countries.^16^

The prevalence of obesity based on BMI did not significantly increase from 2003 to 2008. Using the lower Asian cut-off for obesity (BMI ≥25 kg/m), prevalence increased from 24.4% in 2003 to 26.6% in 2008. During the same period, chronic energy deficiency (CED) decreased from 12.4% in 2003 to 11.6%. The INTERHEART showed that the WHR was a more sensitive index for obesity and that it increased the risk of initial myocardial infarction.^17^ In the Philippines, the prevalence of central obesity, as indicated by WHR, markedly increased among women, from 53.0% in 2003 to 65.6% in 2008, while it nonsignificantly decreased among men during the same period. This was also reflected in WC values, although WC did not significantly differ from WHR in predicting incident CVD events in a meta-regression analysis of prospective studies.^18^ This finding might reflect the effects of unhealthy lifestyles among the general population, in which high caloric intake and fatty food consumption coupled with sedentary lifestyle appear to be increasing.

The increasing prevalence of risk factors known to accelerate cardiovascular disease is likely to increase the prevalence of atherosclerosis-related diseases such as coronary, cerebrovascular, and peripheral arterial diseases. However, the prevalences of these conditions in this study were consistently low and very similar to levels obtained in 2003. In fact, there was a slight decreasing trend in the prevalence of coronary and cerebrovascular disease. Thus, despite the increasing prevalence of risk factors, there may have been an improvement in the resulting diseases, excepting peripheral arterial disease. It is important to emphasize that the present data were obtained from responses to questionnaires. It is possible that respondents did not receive diagnoses of these conditions by medical personnel, which, if true, could have led to underestimation of true prevalence.

In the Philippines, the prevalences of atherosclerosis risk factors, such as hypertension, diabetes mellitus, dyslipidemia, smoking, and central obesity by WHR have generally increased among women. As compared with NNHeS 2008 data from the Philippines, the 2007 Korean NHANES showed that Koreans have a slightly higher mean BMI (23.7), FBG, and HDL-C, but lower SBP, DBP, TC, LDL-C, and TG, as well as downward trends in mean SBP and DBP.^19^ The 2007 Japan National Health and Nutrition Survey showed a downward trend in smoking, an increased prevalence of diabetes, a stable level of obesity in women, and increasing obesity in men.^20^ Based on a study of national, regional, and global trends in SBP from 1980 to 2008, age-standardized mean SBP declined in high-income North America, Australasia, and Western Europe but increased in Oceania, East Africa, and South and Southeast Asia in both sexes.^21^ Age-standardized mean TC changed little during the same period but decreased in the high-income regions of Australasia, North America, and Western, Central, and Eastern Europe. However, mean TC increased in East Asia and Southeast Asia and the Pacific.^22^ Worldwide, mean obesity, as indicated by BMI, has increased, with a few exceptions,^23^ while hyperglycemia and diabetes (DM) are increasing, except in East Asia, Southeast Asia, and Central and Eastern Europe.^24^ The situation in the Philippines is very challenging as the prevalence of several atherosclerosis risk factors increased from 2003 to 2008. These unfavorable circumstances imply that government agencies are lagging in efforts to curtail lifestyle-related risk factors and the resultant diseases, that medical societies are not doing enough to educate the public and control these risk factors, and that the population is either unaware of the consequences of these risk factors or lacks knowledge of how to avoid or control these risk factors. The prevalence of atherosclerosis risk factors is also increasing in neighboring Southeast Asian countries. Dans recently reported that diabetes prevalence correlated with the national affluence in the region, while the prevalences of other risk factors did not. He emphasized that surveillance of chronic noncommunicable diseases and their risk factors needs to be improved in the region and that all branches of government and all sectors of society need to collaborate to create an environment conducive to healthy living.^25^ Perhaps the Philippines and other Asian countries can work toward a similar reduction of these atherosclerosis-related risk factors and diseases.

### Conclusion

In 2008, the true prevalence of hypertension among Filipino adults aged 20 years or older was 20.6%, and the prevalence of diabetes was 3.9% based on FBG, 5.2% based on FBG and history, and 6.0% when the 2-hour post-load plasma glucose level was determined. The prevalence of dyslipidemia was 72.0%, mainly due to the high prevalence of low HDL-C. The prevalence of smoking was 31%. The prevalence of obesity was 4.9% by BMI, and 10.2% in men and 65.6% in women by WHR. Except for smoking, the prevalence of all other measured variables increased as compared with values obtained in 2003. Clearly, additional, better coordinated efforts are necessary from the government and Philippine society.

## ONLINE ONLY MATERIALS

eTables. eTables are available on the journal’s website at http://dx.doi.org/10.2188/jea.JE20110095.
