# Supplementary material for: Prevalence of Atherosclerosis-Related Risk Factors and Diseases in the Philippines
Source: J Epidemiol. 2012 Sep 5;22(5):440–7. doi: 10.2188/jea.JE20110095 (PMC3798639; doi:10.2188/jea.JE20110095)
Supplement: eTables. — eTables are available on the journal’s website at http://dx.doi.org/10.2188/jea.JE20110095. [file je-22-440-s001.pdf]

eTable 1. Number of Respondents and Response Rates for Each Section of Survey (Philippines, 2008)

Variable	Number of Respondents			Total Eligible	Response rate, %
	Total	Men	Women		
Blood pressure	7142	3299	3843	7700	92.8
Fasting blood glucose	6177	2949	3228	7700	80.2
2-hour post-load glucose	4930	2292	2638	7700	64.0
Blood lipids	6276	3003	3273	7700	81.5
Questionnaire	7215	3323	3892	7700	93.7

eTable 2A. Mean Blood Pressure (BP) and Percent Distribution Based on a Single Visit, by Age and Sex (Philippines, 2008)

Age (years)	Mean (mm Hg)		% Distribution by BP Classification*				
			Normal	High Normal	Pre-hypertension	Hypertension	
						Stage I	Stage II
	Systolic BP	Diastolic BP	<120/<80	120-129/ 80-84	130-139/ 85-89	140-159/ 90-99	≥160/ ≥100
Men	123.0	80.8	29.7	28.0	13.2	19.1	10.0
20-29	116.9	77.7	37.2	34.8	11.1	14.0	3.0
30-39	119.3	80.0	32.6	30.2	15.7	16.3	5.2
40-49	124.1	82.9	26.7	25.8	11.7	22.8	13.0
50-59	132.0	85.6	20.1	17.8	13.1	27.7	21.2
60-69	134.4	82.3	18.9	18.9	18.3	24.8	19.1
≥70	139.6	81.2	16.8	19.3	13.8	21.5	28.5
Women	118.7	77.0	46.4	22.6	8.8	14.0	8.2
20-29	108.1	71.7	68.2	22.7	4.3	3.5	1.2
30-39	113.4	75.9	52.8	24.7	8.3	11.7	2.5
40-49	121.3	79.7	38.0	26.3	9.1	16.4	10.1
50-59	130.6	83.2	24.6	18.4	15.2	25.9	15.9
60-69	137.6	82.7	16.8	16.8	13.4	29.0	24.0
≥70	141.1	81.2	13.2	16.7	14.2	26.8	29.0
All	120.7	78.8	38.8	25.0	10.8	16.3	9.0
20-29	112.2	74.5	53.9	28.3	7.4	8.4	2.0
30-39	116.1	77.7	43.7	27.2	11.6	13.8	3.7
40-49	122.6	81.3	32.5	26.1	10.3	19.5	11.5
50-59	131.2	84.2	22.6	18.2	14.3	26.7	18.3
60-69	136.1	82.5	17.8	17.8	15.6	27.1	21.8
≥70	140.2	81.3	14.7	17.8	14.1	24.7	28.8

*Joint National Committee on Detection, Evaluation and Treatment of High Blood Pressure (JNC VII)

eTable 2B. Mean Blood Pressure (BP) and Percent Distribution Based on a Single Visit, by Age and Sex (Philippines, 2003)

Age (years)	Mean (mm Hg)		% Distribution by BP Classification*			
			Normal	Pre-hypertension	Hypertensio n Stage I	Hypertensio n Stage II
	Systolic BP	Diastolic BP	<130/ <85	130-139/ 85-89	140-159/ 90-99	≥ 160/ ≥100
Men	123.1	78.4	34.2	41.5	16.1	8.1
20-29	118.8	75.3	35.4	51.6	11.5	1.5
30-39	118.9	77.6	43.4	37.5	14.3	4.8
40-49	124.2	82.0	30.3	40.1	18.9	10.7
50-59	131.4	82.6	25.1	33.8	22.4	18.7
60-69	136.5	80.3	19.3	36.4	23.7	20.5
≥70	141.4	76.9	17.9	31.3	25.8	25.0
Women	119.7	75.3	48.9	30.4	12.4	8.4
20-29	108.1	69.3	72.9	23.4	2.8	0.9
30-39	111.7	73.4	59.9	32.0	6.6	1.5
40-49	121.0	78.9	42.0	34.9	14.2	8.9
50-59	131.9	81.1	25.4	34.9	23.9	15.8
60-69	138.1	79.5	21.2	31.8	25.2	21.8
≥70	146.3	77.5	13.9	26.8	27.1	32.2
ALL	121.5	76.8	41.4	36.1	14.3	8.2
20-29	114.0	72.6	52.2	39.0	7.6	1.2
30-39	115.6	75.8	50.8	35.1	10.8	3.3
40-49	122.7	80.5	36.0	37.5	16.6	9.8
50-59	131.7	81.8	25.3	34.4	23.3	17.0
60-69	137.4	79.8	20.4	33.8	24.6	21.2
≥70	144.3	77.3	15.4	28.5	26.6	29.4

*Joint National Committee on Detection, Evaluation and Treatment of High Blood Pressure (JNC VII)

eTable 3. Prevalence of dyslipidemia (2003 and 2008)

Lipid parameters	Prevalence, %	
	2003	2008
Total cholesterol <4.14 mmol/L	28.8	28.3
Total cholesterol ≥6.20 mmol/L	8.5	10.2
LDL-C ≥4.14 mmol/L	11.7	11.8
HDL-C <1.03 mmol/L	54.2	64.1
Triglycerides ≥2.26 mmol/L	8.7	14.6
Dyslipidemia	62.5	73.1

Abbreviations: LDL-C, low-density lipoprotein cholesterol; HDL-C, high-density lipoprotein cholesterol.

eTable 4A. Mean and Percent Distribution of BMI Among Adults Aged 20 Years or Older, by Sex, Age, and WHO BMI Classification (Philippines, 2008)

Sex / Age Group	Mean BMI	BMI, %			
		Chronic Energy Deficient	Normal	Overweight	Obese
		<18.5	18.5-24.9	25.0-29.9	≥30.0
Males	22.6	10.1	66.9	19.3	3.7
20-29	21.9	11.0	73.7	13.0	2.2
30-39	23.3	5.8	65.7	23.6	4.9
40-49	23.2	6.6	65.3	23.1	5.0
50-59	22.8	10.7	63.7	21.4	4.2
60-69	21.8	17.7	63.0	16.8	2.4
≥70	20.8	26.5	61.9	10.4	1.2
Females	23.1	12.9	57.1	23.5	6.6
20-29	21.8	17.0	66.1	13.2	3.6
30-39	23.6	8.7	59.0	25.1	7.3
40-49	24.0	7.8	54.8	29.1	8.4
50-59	23.6	12.1	52.3	27.6	8.0
60-69	22.8	17.7	53.1	22.8	6.4
≥70	21.4	27.4	52.4	17.1	3.2
All	22.9	11.6	61.8	21.4	5.2
20-29	21.9	13.7	70.3	13.1	2.9
30-39	23.4	7.2	62.3	24.4	6.1
40-49	23.6	7.2	59.7	26.3	6.8
50-59	23.2	11.5	57.5	24.8	6.3
60-69	22.4	17.7	57.5	20.1	4.6
≥70	21.2	27.0	56.2	14.4	2.4

Abbreviations: BMI, body mass index; WHO, World Health Organization.

eTable 4B. Distribution of body mass index (BMI), based on WHO criteria for overweight and obesity, by age and sex (Philippines 2003)

Age, y	Chronic energy-deficient (<18.5)	Low normal (18.5 -19.9)	Normal (20.0 -24.9)	Overweight (25.0 - 29.9)	Obese (≥30.0)
Men					
20-29	11.0	14.0	60.6	12.9	1.5
30-39	7.3	14.2	53.8	20.5	4.1
40-49	9.7	11.1	48.9	26.0	4.1
50-59	12.4	13.1	45.8	24.1	4.7
60-69	23.6	12.3	46.6	15.1	2.3
≥70	28.1	22.1	41.7	7.2	0.8
Subtotal	11.1	13.6	53.3	18.8	3.2
Women					
20-29	18.4	17.4	47.3	11.5	5.3
30-39	8.0	11.2	54.8	19.9	6.1
40-49	11.4	7.0	43.4	30.9	7.2
50-59	10.3	7.6	44.7	26.4	11.0
60-69	19.5	14.3	39.6	22.0	4.6
≥70	28.1	16.7	37.2	14.7	3.3
Subtotal	13.7	12.1	47.1	20.5	6.6
Overall	12.4	12.9	50.2	19.6	4.8

Abbreviations: BMI, body mass index; WHO, World Health Organization.

eTable 5. Prevalence (%) of Metabolic Syndrome and its Components (2003 and 2008)

	2003	2008
Elevated BP (≥130/80 mm Hg)	33.3 (1.0)	39.3 (0.70)
Elevated glucose (≥5.55 mmol/L)	7.1 (0.4)	12.2 (0.62)
WC (M ≥90, F ≥80 cm)	26.1 (0.96)	29.8 (0.72)
HDL-C (M <1.03 mmol/L, F <1.30 mmol/L)	70.2 (1.0)	80.3 (0.62)
Triglycerides (≥2.26 mmol/L)	20.6 (0.6)	30.1 (0.67)
Metabolic syndrome	18.6 (17.0-20.2)	27.4 (0.67)

Abbreviations: BP, blood pressure; WC, waist circumference; HDL-C, high-density lipoprotein cholesterol.
